# Delivering health knowledge and wisdom from the hills and hollows of Appalachia

**DOI:** 10.13023/jah.0101.01

**Published:** 2019-04-30

**Authors:** F. Douglas Scutchfield, Erin N. Haynes, Robert M. Shapiro, Charlotte S. Seidman

**Affiliations:** University of Kentucky, scutch@uky.edu; University of Kentucky, erin.haynes@uky.edu; University of Kentucky, Chandler Medical Center Library, shapiro.rm@uky.edu; Seidman Writing, charlotte.seidman@uky.edu

**Keywords:** Appalachia's health, improving the health of the region

## Abstract

There is knowledge in the pages of Appalachia’s hills. This journal is positioned to find and publish those translations. It grows from a need to provide an outlet for scholarship about Appalachia’s health so that knowledge, and occasionally wisdom, is shared with those who care about and are committed to improving the region’s health.

But there are those who learn what is told here By convolutions of earth, by time, by winds, The water’s wearings and minute shapings of man. They have struck pages with the large print of knowledge, The thing laid open, the hills translated.

*River of Earth* by James Still

As Still suggests in his poem, there is knowledge in the pages of Appalachia’s hills. This journal is positioned to find and publish those translations of which Still speaks. It grows from a need to provide an outlet for scholarship about Appalachia’s health so that knowledge, and occasionally wisdom, is shared with those who care about and are committed to improving the region’s health. To accomplish that translation, we have established this journal. This is the first issue of the first volume of what we hope and believe will be the first of many volumes that will “print the knowledge” of Appalachian health.

We were inspired to develop this journal based on the continued concern of the poor health of Appalachian people and the lack of a journal dedicated to share the scientific research conducted to improve their health. Recent research demonstrates that, while there have been improvements in the health of those who live in these hills, there remains a substantial disparity in health status when compared to the nation’s health. This disparity is the result of inequities that exist in the region. We hope to look at those disparities and inequities so that we can better understand the nature of health problems and the positive impact of the programs that address them.

There is a body of knowledge about Appalachia’s health and how it might be addressed that does appear in the published literature; for example, in the last 5 years there have been about 15,000 articles with the keyword Appalachia listed in PubMed, yet these are scattered throughout the health literature, and there is currently no single journal dedicated to scholarly works related to Appalachian health. Until today there has not been an easy and appropriate outlet for research specifically related to Appalachia’s health problems and programs. This journal fills that gap.

The *Journal of Appalachian Health* looks at health and health programs as a concept that is influenced by a broad set of health determinants. These determinants include behavior, environment, socioecological conditions, and available health services. If we seek healthy and thriving communities in Appalachia, then consideration of health must include understanding these determinants and how to appropriately address them. To do that we must open the literature to individuals and organizations that are not traditionally considered as “health” related. They can, in many cases, be the source of solutions to the inequities that reflect Appalachia’s health. If that is so, we must assure scholarship in areas such as housing, food security, poverty, and education, as well as health care, as grist for our journal.

This journal is supported by the Robert Wood Johnson Foundation and the partners listed on our masthead. This allows us to offer the *Journal of Appalachian Health* without requiring subscriptions or author publication fees. It is free to both authors and readers. The sponsors and the advisory committee came together in a strategic planning session that resulted in the mission statement summarized in our tag line: A Healthy and Thriving Appalachia. We have a series of strategic goals and specific objectives for the journal and will be tracking those as we proceed, to make this the “go-to” journal for anyone committed to the creation of a healthy and thriving Appalachia!

The journal is open access, online, and peer-reviewed. We anticipate and are pointing toward assuring its indexing in NLM’s indexing service, as well as others, to allow us to assure that the content is readily available for reference and citation. Our primary articles will be research reports, and we have adopted the style for those based on CDC’s *Morbidity and Mortality Weekly Report* to make them as reader-friendly as we can get them. They are purposely short, and we will make available additional supplemental material so that we can keep research as reader-friendly as possible. We hope that this will be attractive to those who are not part of a health organization but who are involved in ameliorating conditions that contribute to health status.

We will be including other material that we hope will add to the understanding of Appalachian health. For example, we include a section called “Voices from the Hollows” to provide a narrative description or discussion from those who are actively engaged in Appalachian health can contribute in a qualitative and not quantitative way. This will allow us to assure that we cover broad content related to the socioecologic contributors to Appalachia’s health status. We will be providing editorials on some the research articles to help clarify their message and add some observations and thoughts. We will also be publishing occasional invited commentary from senior scholars on Appalachian health. We anticipate an occasional “Report from the Fed” section where our federal colleagues and partners can bring readers abreast of important information from their activities and policies. Some of these features are in our inaugural issue, but some will come as we proceed with the journal. We welcome letters to the editor and, in fact, would love to hear from our readers how we can increase the value of the journal.

So, you now hold the new *Journal of Appalachian Health* in your electronic hands, dear reader. We want to assure that the journal is as useful to you as it can possibly be and will be looking for suggestions from you about how this journal can contribute to addressing the health of the population of Appalachia. As we move forward, we will also be reaching out to readers to ascertain their satisfaction and thoughts and ideas. Let us hear y ’all’s ideas and comments.

